# Event-Based, Timescale Invariant Unsupervised Online Deep Learning With STDP

**DOI:** 10.3389/fncom.2018.00046

**Published:** 2018-06-14

**Authors:** Johannes C. Thiele, Olivier Bichler, Antoine Dupret

**Affiliations:** CEA, LIST, Gif-sur-Yvette, France

**Keywords:** spiking neural network, STDP, unsupervised learning, event-based learning, deep learning, online learning, digit recognition, neuromorphic engineering

## Abstract

Learning of hierarchical features with spiking neurons has mostly been investigated in the database framework of standard deep learning systems. However, the properties of neuromorphic systems could be particularly interesting for learning from continuous sensor data in real-world settings. In this work, we introduce a deep spiking convolutional neural network of integrate-and-fire (IF) neurons which performs unsupervised online deep learning with spike-timing dependent plasticity (STDP) from a stream of asynchronous and continuous event-based data. In contrast to previous approaches to unsupervised deep learning with spikes, where layers were trained successively, we introduce a mechanism to train all layers of the network simultaneously. This allows approximate online inference already during the learning process and makes our architecture suitable for online learning and inference. We show that it is possible to train the network without providing implicit information about the database, such as the number of classes and the duration of stimuli presentation. By designing an STDP learning rule which depends only on relative spike timings, we make our network fully event-driven and able to operate without defining an absolute timescale of its dynamics. Our architecture requires only a small number of generic mechanisms and therefore enforces few constraints on a possible neuromorphic hardware implementation. These characteristics make our network one of the few neuromorphic architecture which could directly learn features and perform inference from an event-based vision sensor.

## 1. Introduction

Deep Learning has in recent years become one of the most popular and powerful machine learning methods. In particular, convolutional neural networks (CNNs) in combination with the backpropagation algorithm (Rumelhart et al., 1986) provide the state-of-the-art in image recognition and deep neural networks are employed in an ever increasing number of demanding applications (LeCun et al., 2015).

Although the main criticism for current deep learning methods comes mainly from their lack of biological plausibility, we think that even from an engineering perspective, spike-based models could offer advantages over standard, frame-based deep learning architectures, in particular for energy critical applications. The main cause of the potential energy efficiency of spike-based systems is the absence of multiplication operations (if implemented in the corresponding hardware) and their event-driven nature, which processes information only if it is provided to the network externally. This stands in contrast to frame-based systems, which depend on a constant and energy-consuming high-frequency stream of images to detect changes in the environment.

Most recent spiking deep network models focus on the energy efficiency aspect and have shown the ability to train deep networks with spikes with increasing precision. A large number of recent studies have focused on the implementation of the successful backpropagation algorithm on a spike-based architecture (see for instance Lee et al., 2016; O'Connor and Welling, 2016; Neftci et al., 2017 for networks with rate coding and Liu et al., 2017; Mostafa, 2017; Wu et al., 2017 for spatio-temporal coding). Others have derived even more general gradient-descent update rules for spiking neural networks (Huh and Sejnowski, 2017; Zenke and Ganguli, 2017). Their preliminary results show good performance on simple benchmark tasks such as the MNIST dataset and the potential to learn complex spatio-temporal tasks. The spike-based unsupervised deep architectures of Kheradpisheh et al. (2017), Panda et al. (2017), and Tavanaei and Maida (2017) use a simplified unsupervised STDP rule (Bi and Poo, 1998) in combination with a winner-takes-all (WTA) mechanism to extract hierarchical features in CNN-like architectures. This enables them to process large scale natural images of natural objects (for instance human faces). The best classification results for this type of network are provided by Kheradpisheh et al. (2017) and Tavanaei and Maida (2017), who use however a supervised support vector machine classifier on the extracted features to evaluate final classification performance. A supervised spike based classifier based on reinforcement learning was tested in the same framework by Mozafari et al. (2017). Yousefzadeh et al. (2017) demonstrated the possibility to extract simple features with competitive STDP in a FPGA implementation of a spiking neural network.

Despite the success of these recent approaches, we believe that they only partially exploit the potential strength of an event-based learning approach. Almost all existing spike-based deep networks are in a sense still based on the frame-based learning paradigm, where classification performance is optimized over a given, possibly fully labeled dataset with well separated training examples of fixed presentation time. In contrast to this previous work, we will focus on an event-based online learning setting, i.e., a scenario where the network receives a continuous and asynchronous stream of unlabeled event-based data from which it extracts features and performs approximate online classification. This requires a system which is able to learn features from a constantly changing scene with objects appearing at different timescales. Since supervised models fundamentally rely on labeled training data to learn features, they are unsuitable for an application where there is no clear relationship between the input and a certain label. But also the aforementioned unsupervised architectures are trained in a greedy-layer wise fashion, where each layer is greedily optimized for unsupervised feature extraction on the full data set before the next layer is trained in the same way. This takes away the possibility to perform approximate inference already during the learning process. It also makes it impossible to use the output of higher layers to influence the features learned in the layers below, making it for instance difficult to combine the network with tools such as reinforcement learning or any other mechanism which involves feedback from higher layers to lower ones and therefore enables complex multi-layer representations. Additionally, it would be desirable not to depend on floating-point-based classifier (such as a SVM) in the top layer since it violated the spike-based learning paradigm. It also leaves it kind of unclear how much of the classification performance is due to the quality of the extracted features and how much due to the learned parameters of the classifier.

We will try to approach several aspects of the above mentioned problems by taking a bio-inspired engineering approach. This means that our main concern will be the potential to implement our network on an event-based neuromorphic hardware platform, and we will considering biological plausibility mainly where it could offer potential benefits to our architecture. By introducing a mechanism to decouple winner-takes-all (WTA) dynamics from spike propagation, all layers of our network can be trained simultaneously. This enables us to perform approximate inference during the learning process. Furthermore, we introduce a learning mechanism which removes any notion of absolute time from our network such that all spike times are measured relative to the dynamics of postsynaptic spikes, making our network fully event-based. In addition, we show that it is possible to train the network without providing any information about the structure of the input data (such as the number of classes) and treating the training set as a continuous stream of event-based input. This also includes implicit information, such as the duration of image presentation. We demonstrate the high convergence speed and robustness of learning with respect to some of the typical problems which might occur in an online learning setting. Despite these constraints, our network yields a test accuracy of (96.58±0.16%) on the spike-converted MNIST benchmark after a single presentation of the training set, using only a single neuron spike count classifier. This is the highest reported score so far for a network where all connections up to the final feature are trained solely with unsupervised STDP and demonstrates the high specificity of neurons in the top layer.

## 2. Methods

### 2.1. Network architecture

**Figure 1:**
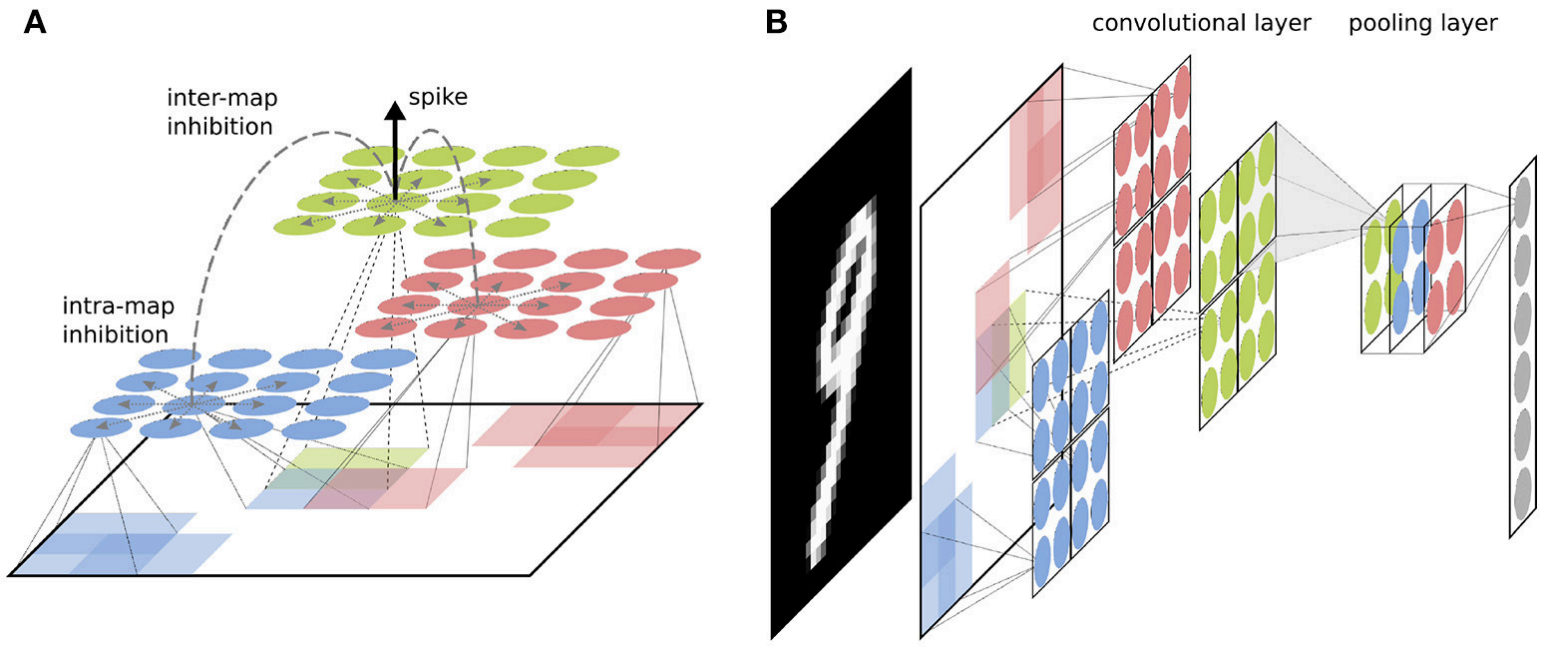
**(A)** Basic structure of a convolutional layer with inter-layer WTA. Each feature map (shown in different colors) detects features in an overlapping manner over the full image. If a neuron spikes in a feature map, it inhibits neurons in all neural maps around a small neighborhood of its position. **(B)** Visualization of a convolutional layer preceeded by a pooling layer. Neurons in the next layer connect to the pooling layers as they would to a two dimensional image, with the difference that weights extend to a third dimension through all feature maps. This way the layer aggregates information from all features maps simultaneously.

The fundamental setup of our network is similar to other competitive convolutional architectures trained with STDP (such as Kheradpisheh et al., 2017; Mozafari et al., 2017; Panda et al., 2017; Tavanaei and Maida, 2017). The network parameters used for all simulations can be found in Table 1. We use two convolutional layers with a varying number of neural maps depending on the experiment. If not stated otherwise, all simulations used by default 16 neural maps in the first and 32 maps in the second convolutional layer, as well as 1,000 neurons in the fully connected top layer (see Figure **3**). Both convolutional layers use a filter size of 5 × 5 with a stride of 1. The neuron firing threshold values are 8 (first convolutional layer), 30 (second convolutional layer), and 30 (fully connected layer) respectively. There is a intra-map WTA inhibition mechanism which inhibits all other neurons in a neural map at different position as soon as one neuron in the map releases a spike (see Figure 1). Inhibition is performed by resetting the integration variables and reference time of the inhibited neurons (such that spike times are always measured with respect to the last reset of the integration variable). This mechanism prevents a single map from dominating the learning competition at all position of an input image by learning a feature which is too general. The second inhibitory mechanism acts in an identical way between neural maps (inter-map) and inhibits neurons neurons in a small neighborhood of the position of the spiking neuron in all neural maps. This neighborhood will typically be chosen such that all neurons whose filters overlap with the filter of the firing neuron will be inhibited (in our case the two next neighbors). This competitive mechanism is essential to diversify the features learned by different neural maps.

**Table 1:** Network parameters used for the simulations.

**Description**	**Parameter**	**Value**
**CONV 1**		
Threshold STDP	*T*_inh, STDP_	8
Threshold propagation	*T*_inh, prop_	8
Inter-map inhibition radius STDP (in nearest neighbors)	*r*_inh, STDP_	2
Inter-map inhibition radius propagation (in nearest neighbors)	*r*_inh, prop_	0
Ration LTP vs. LTD	α_+_/α_−_	−8
Filter size	*d*_filter_	5
Stride	*s*_filter_	1
Initial weights with STD (normally distributed)	*w*_init_	0.8 ± 0.1
**CONV 2**		
Threshold STDP	*T*_inh, STDP_	30
Threshold propagation	*T*_inh, prop_	30
Inter-map inhibition radius STDP (in nearest neighbors)	*r*_inh, STDP_	2
Inter-map inhibition radius propagation (in nearest neighbors)	*r*_inh, prop_	0
Ration LTP vs. LTD	α_+_/α_−_	−8
Filter size	*d*_filter_	5
Stride	*s*_filter_	1
Initial weights with STD (normally distributed)	*w*_init_	0.8 ± 0.1
**FC**		
Threshold STDP	*T*_inh, STDP_	30
Threshold propagation	*T*_inh, prop_	30
Ration LTP vs. LTD	α_+_/α_−_	−8
Initial weights with STD (normally distributed)	*w*_init_	0.67 ± 0.1

*An inhibition radius of 0 indicates that only neurons at exactly the same position in other maps are inhibited*.

After each convolutional layer, the network performs a pooling operation over non-overlapping windows of size 2 × 2 in each neural map to reduce the dimensionality of the input. In contrast to the architecture introduced in Kheradpisheh et al. (2017), where a form of max-pooling is performed which only propagates the first spike in a pooling window, our pooling layer propagates all spikes which are emitted in the pooling window. This is necessary if the network should be defined independent of the input timescale, since else we would have to define a point at which the pooling neuron is unlocked again (which is usually done when a new example is presented). Additionally, this allows us to propagate a more flexible number of spikes to the following layers, while reducing the size of the visual input. In our implementation, the pooling neurons are not actual neurons since they simply propagate all spikes from a certain input region, but in principle the pooling neurons could be replaced by more complex neuron model which has a more specific selectivity or a threshold value. The basic module of convolutional layer followed by pooling layer can in principle be arbitrarily copied to form a multi-layer deep network.

Similar to frame-based CNNs, the convolutional layers are followed by a fully connected layer, which is trained with the same STDP mechanism as the convolutional layers. It merges the features from all positions and features maps to learn global, position independent representations of the classes present in the input data. This distinguishes our architecture from the other multi-layer competitive architectures mentioned above and more similar in spirit to the single layer networks of Diehl and Cook (2015) and Querlioz et al. (2011). This type of representation enables us to obtain spikes in the last layer which are direct indicators of the class the network detects.

### 2.2. Dual accumulator neurons

To make our architecture suitable for online learning, several paradigms have to be reconsidered which were present in previous work. The main problem when training all layers simultaneously comes from the WTA mechanism which is used in lower layers. Although inhibition is necessary to diversify the learned features in each layer, it significantly reduces the number of spikes which are emitted by the neurons and prevents spikes from different maps at the same input position. This limits the amount of information which is received by the higher layers and also prevents the propagation of spike codes which are a combination of several features maps. In the layer-wise training approach, this problem is resolved by disabling inhibition after the layer has been trained and then training the next layer on the output of all lower layers.

**Figure 2:**
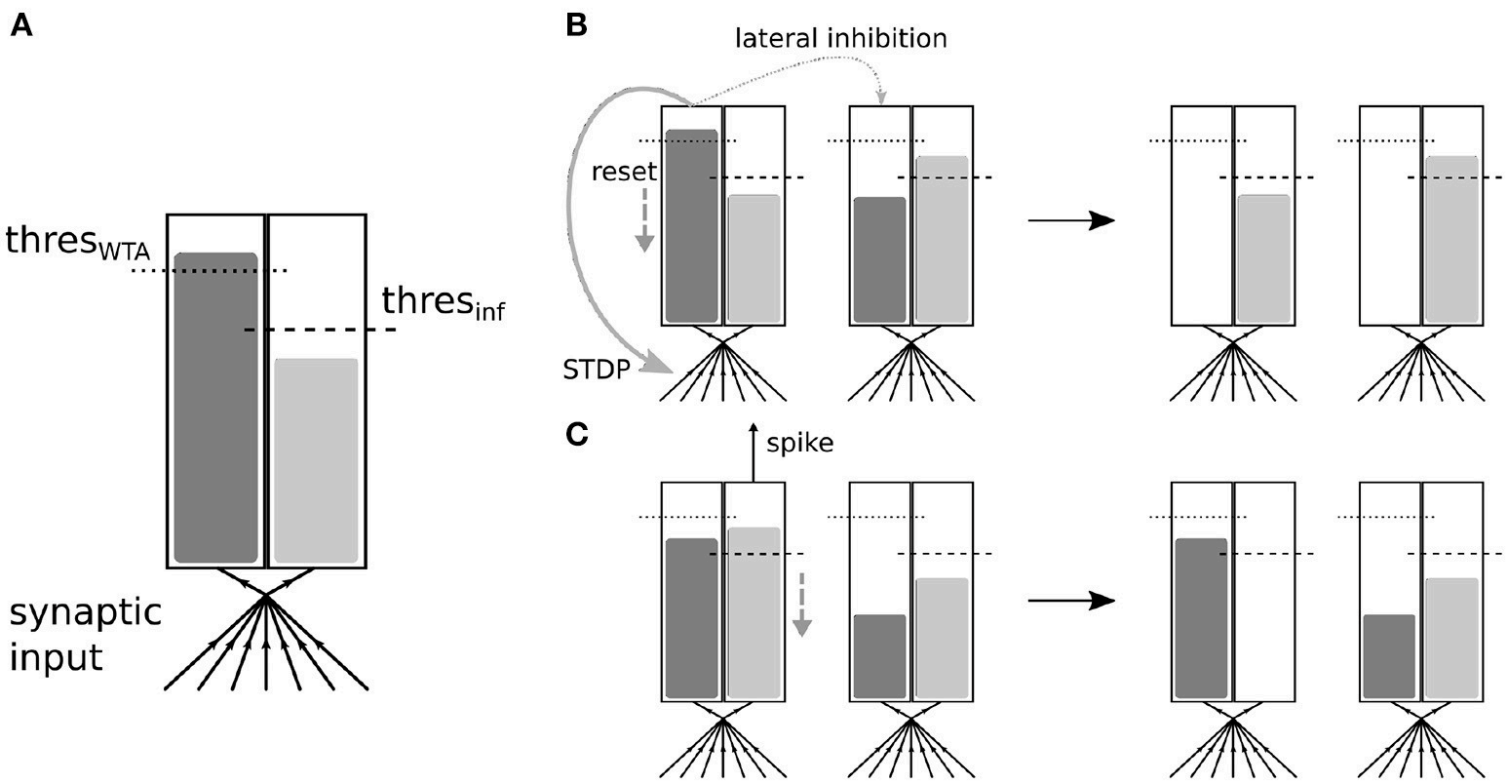
**(A)** Schematic description of the multi-threshold neuron. The neuron integrates all inputs it receives simultaneously in two accumulators, one for STDP triggering and WTA, and the second one for inference (i.e., spike propagation). **(B)** Interaction between two neurons coupled via lateral inhibition. If the integration in the WTA accumulator reaches the threshold value, no actual spike is propagated. However, the neuron will behave as if it had spiked, which includes a reset of its integration value, a triggering of STDP and WTA lateral inhibition which affect only the WTA accumulators of the other neuron (by reseting them). **(C)** If integration reaches the threshold value in the inference accumulator, a spike will be propagated and integration will be reset. The inference accumulators of other neurons are not necessarily affected.

In our work, we take a different approach, which enables us to keep lateral inhibition and still obtain sufficient spikes to train higher layers. We introduce a second integration accumulator in the neuron which receives the same input, but is not affected by lateral inhibition and whose “spikes” do not trigger STDP updates (see Figure 2). This corresponds to a separation of the competitive learning dynamics from the inference dynamics. Since the inference accumulator receives the same feed-forward input as the learning accumulator, the spiking of the inference accumulator is still a good representation of how well the input matches the receptive field of the neuron. Both accumulators can in principle have different threshold values. By tuning the threshold of the inference accumulator, the firing rate of the neurons can be adjusted without affecting the learning mechanism. More spikes will generally lead to faster convergence in higher layers and higher inference precision, but also require more computational resources.

Additionally, our experiments show that it can still be beneficial for learning in higher layers to enable some inter-map inhibition between inference neurons. This mostly depends on the need to have several features contributing to the emitted spike code at a particular position or only the most salient feature. It therefore enables us to smoothly switch between a one-hot feature representation, where only one feature can be active at a given position, and a more continuous representation, where multiple features can contribute partially. As shown by Maass (2000), lateral inhibition in neural circuits can have powerful computational capabilities and depriving the inference accumulators of this mechanism could therefore have a detrimental effect on the representational capabilities of the network. The dual accumulator neuron allows us to treat the competitive aspects of learning and coding independently.

### 2.3. Online learning constraints

An additional objective of our approach is to make the system completely event-driven and remove any notion of an absolute time scale. In particular, this means that the network should not be given any explicit or implicit knowledge about the timescale of the input data, as for example when a training example is exchanged for the next one. This has several consequences for the setup of our network:

- No leakage currents: we use a simple integrate-and-fire neuron
- No refractory periods: the firing of a neuron is solely driven by its integration, spike, and reset dynamics
- No inhibitory refractory periods: WTA inhibition directly reduces the integration variable of other neurons to prevent them from firing
- No reset of neuron values when a new training example is presented, since this provides the network with implicit information about the training set
- No restriction of neuron firing which relates to the presentation of a training examples, for instance the restriction of neuron firing to once per training example
- No homeostatic mechanisms which use implicit knowledge of the training set statistics, for example constraining the neurons in the last layer to have equal firing rates
- No additional pre-processing of stimuli (for instance input normalization). The network has to be able to deal with strongly varying numbers of spikes for each stimulus.

These changes allow our network to be only driven by the timescale of its input, which can in principle even change during the learning process, without affecting the network dynamics. The only requirement for the network to be able to learn from input spikes is that sufficient spikes are produced in the network to trigger the STDP mechanism. Our network therefore fully embraces the paradigm of asynchronous event-based processing without depending on a clock-based mechanisms.

### 2.4. Learning rule

As weight update rule, we use a version of the STDP learning rule introduced in Querlioz et al. (2011). Every time a neuron triggers a postsynaptic spike, its weights are updated as follows:

(1) $$\Delta w=\{\begin{array}{ll} \;\alpha_{+}\exp\left(-\beta_{+}\frac{w-w_{\text{min}}}{w_{\text{max}}-w_{\text{min}}}\right) & \text{if }t_{\text{post},\text{last}}<t_{\text{pre}}<t_{\text{post}}\\ \;\alpha_{-}\exp\left(-\beta_{-}\frac{w_{\text{max}}-w}{w_{\text{max}}-w_{\text{min}}}\right) & \text{otherwise} \end{array}$$

with learning rates α_+_>0 and α_−_ < 0 and damping factors β_−_, β_+_≥0. Our experiments showed that this learning rule works best if we use a rather strong damping of β_+_ = 3 for the LTP (long-term potentiation) term and no damping (β_−_ = 0) for LTD (long-term depression). Additionally, we constrain the weights to be in the range [0, 1]:

(2) $$\Delta w=\{\begin{array}{ll} \;\alpha_{+}\exp(-\beta_{+}w) & \text{if }t_{\text{post},\text{last}}<t_{\text{pre}}<t_{\text{post}}\\ \;\alpha_{-} & \text{otherwise} \end{array}$$

In contrast to Querlioz et al. (2011) we do not use an STDP time window to decide between LTP and LTD. Every time a postsynaptic neurons spikes at time *t*_post_, synapses coming from neurons which spiked since the last postsynaptic spikes at time *t*_post, last_ are potentiated, and depressed if they did not spike. Since the membrane potential of the postsynaptic neuron is reset after each spike, this ensures that only neurons which directly contributed to the current postsynaptic spike are potentiated. A similar learning mechanism was used in Kheradpisheh et al. (2017), however with a different weight dependence. The main reason for adding the exponential weight dependence is the tendency of STDP to converge too quickly to the minimal and maximal weight values. As argued in Querlioz et al. (2011), the computationally expensive exponential function in our learning rule could be implicitly implemented by the device physics of a memristive synapse. Besides these practical considerations, our experiments show that the architecture does not depend too strongly on the exact details of the STDP rule and also works with minor performance losses with a simpler version, which does not include the exponential weight dependency.

The rule (2) is qualitatively very similar to the optimal STDP rule for stochastic neurons introduced by Nessler et al. (2013) and explored in more detail by Habenschuss et al. (2012) and Habenschuss et al. (2013). This similarity has been pointed out already by Querlioz et al. (2015) in their analysis of rule (1) in the context of a memristive hardware implementation. Although the optimality condition does not strictly apply here due to the non-stochastic firing dynamics of the spiking neurons, it is interesting that this very similar rule seems to empirically yield the best results in our network. Tavanaei et al. (2016) showed that this learning rule has a probabilistic interpretation and converges to weights which represent the log odds of the firing probability of the neuron. Additionally, we observed that only the ration of α_+_ and α_−_ seems to influence the quality of the learned features (with the absolute values still guiding the speed of learning). A similar behavior has been observed in Querlioz et al. (2015) and Kheradpisheh et al. (2017) which indicates that this could be a general feature of this class of simple postsynaptic STDP learning rules. Another interesting feature is that the same learning rule with the same ratios can be applied to all convolutional layers, as well as the fully connected layer at the top of the network. Only the magnitude of the factors has to be adapted to account for the different learning speed of every layer, which is a consequence of the different number of spikes which are triggered in each layer. Since our rule is driven by postsynaptic spikes, the number of spikes caused in a layer will strongly influence the learning speed. We observed that the ration of the rates seems to depend strongly on the threshold values of the neurons. Since our learning rule only distinguishes between synapses which have spikes and those who have not, a high threshold value will allow more synapses to contribute before the postsynaptic spike is triggered. To obtain a feature which is sensitive to spikes from those synapses which actually spike more often, we will have to use a learning rate with rather strong depression, since there will be statistically only a few situations where a synapse did not spikes at all and is therefore subject to LTD. The inverse is true for a low threshold value, where only a few synapses will contribute on average to a postsynaptic spike and depression is therefore the more common scenario, which is why it should be rather weak. For our choice of parameters, we found that a ratio of α_+_ = −8α_−_ works best in practice. This leads to a learning rule with high LTP for small weight values which is strongly damped if the value increases. Already for a weight value of *w* = −1/β_+_log(−α_−_/α_+_)≈0.7, depression becomes stronger than potentiation, which will effectively prevent weights from saturating. This behavior is important for the online learning capabilities of our architecture, since a saturation of weights could prevent the system from learning on additional examples. At the same time, the learning rule stays highly sensitive even if *w* is close to zero, which allows the system to adapt to changing input statistics.

In contrast to Querlioz et al. (2011) and Diehl and Cook (2015), our network does not use a homeostatic mechanism to adjust the firing rates of the neurons. Although such a mechanism was shown to greatly improve performance on this task for one-layered networks, we decided against such a mechanism since it makes implicit assumption about the statistics of the training set. For instance, enforcing equal firing rates for neurons over long timescales, which is a typical objective for homeostatic mechanisms, imposes that the features represented by each neuron occur approximately equally often. This is in general not true for an online learning task and in particular the intermediate level features learned by the convolutional layers do not have equal probabilities of occurrence.

### 2.5. Timescale invariant STDP learning

Intuitively, we can understand the STDP mechanism the following way: the threshold of the neurons of a layer describes a type of implicit timescale, which is the time until one of the neurons has integrated enough information to emit a spike. All neurons which are connected to this neuron by inhibitory connections are subject to a reset of their integration variable and their relative time scale. As long as the input dynamics change much slower than this implicit timescale, there will be a consistent causal relationship between a certain synaptic input and the response of a neuron. Since the time until the threshold is reached depends only on the total integrated input signal, this implicit time scale is directly set by the timescale of the input. If spikes arrive very rapidly, the threshold will be reached in a short time. If spikes arrive rather slowly, also the absolute time until the threshold is reached becomes longer. In a model which uses a leakage current or refractory times, these times would have to be adjusted to the timescale of the input signal. Since our STDP rule depends only on the relative timing of postsynaptic spikes, and therefore on this implicit timescale, we are able to use the causality reinforcement properties of STDP without defining an absolute reference time.

### 2.6. Input encoding

The network is trained on a *single* randomly ordered presentation of the full MNIST dataset of 60,000 digits (i.e., no digit was shown twice to the network). No pre-processing is performed. Each images is converted to noisy periodic spike trains with mean firing rates proportional to the absolute value of the pixel values, which are converted to lie in the range [0, 1]. Each spike train is randomized by drawing the mean firing rate from a Gaussian distribution and additionally multiplying the constant inter-spike interval length with a random number in the range [0, 1] every time an event is created. However, our experiments show that the feature learning does not depend significantly on this particular conversion of the images to firing rates, as long as the firing rates grow approximately with pixel intensity. In the standard experiment, all images are presented for a fixed time to the network. Note that the timescale here is only necessary for the spike generator and therefore only influences the number and statistics of the spikes emitted by each training example. The processing of the network only requires the relative timing of spikes. If not stated otherwise, the presentation time is set such that each training example emits approximately between 1,400 and 3,500 spikes in total, depending on the average value of all pixels in the image.

### 2.7. Testing procedure

After training, the 60,000 digits of the training set are used to label the neurons and in a second pass the test performance is evaluated on all 10,000 test images. Since our mechanism is unsupervised, we still need a simple classifier to assign to each neuron in the last layer the label of its preferred class. This is simply done by presenting each training image to the network and assigning to each neuron the corresponding label if it is the neuron with the highest response for this image. The preferred label of a neuron is the label which was most often assigned to it via this process. For inference, we only check if the preferred label of the neuron which fired the most during presentation of the test sample corresponds to the label of presented image. If this is the case, the classification is considered as correct. This mechanism can be seen as a minimal classifier which uses only the prediction of the most active neuron for classification. It is therefore a valid measure for the class specificity of the neurons in the last layer. In particular, it does not influence the learned features themselves, but only their interpretation. This distinguishes it for example from a more complex classifier such as an SVM, which performs a classification based on a weighted combination of neuron outputs of the last convolutional layer, with parameters trained in a supervised fashion. Our approach corresponds to the more realistic unsupervised learning scenario where generally much fewer labels than training instances are available and therefore the labels can only be used to assess the networks performance, but cannot be used for feature learning. An analysis of how the number of labels affects the classification performance can be seen in Figure **6**. We can see that a few labels are sufficient for approximate classification, but using more labels of the training set further improves the performance. This makes it possible to train the network without providing many labels and still use them to improve the classification in the final layer if they become available. We restricted ourselves in this work to a simple classifier to obtain a meaningful measure of the quality of the top level features. Depending on the complexity of the hardware implementation, a more complex supervised classifier could be used for the top layer to improve inference performance (see for example Stromatias et al., 2017 for a supervised classifier trained with gradient descent on spike histograms).

### 2.8. Event-based simulation

All simulations were performed with a modified version of the N2D2 open source deep learning library by Bichler et al. (2017), using the embedded event-based spiking neural network simulator.

## 3. Results

In this section, we present the main results of our simulation and investigate several properties of the architecture which could be of interest for an unsupervised online learning application.

### 3.1. Feature learning and inference performance

**Figure 3:**
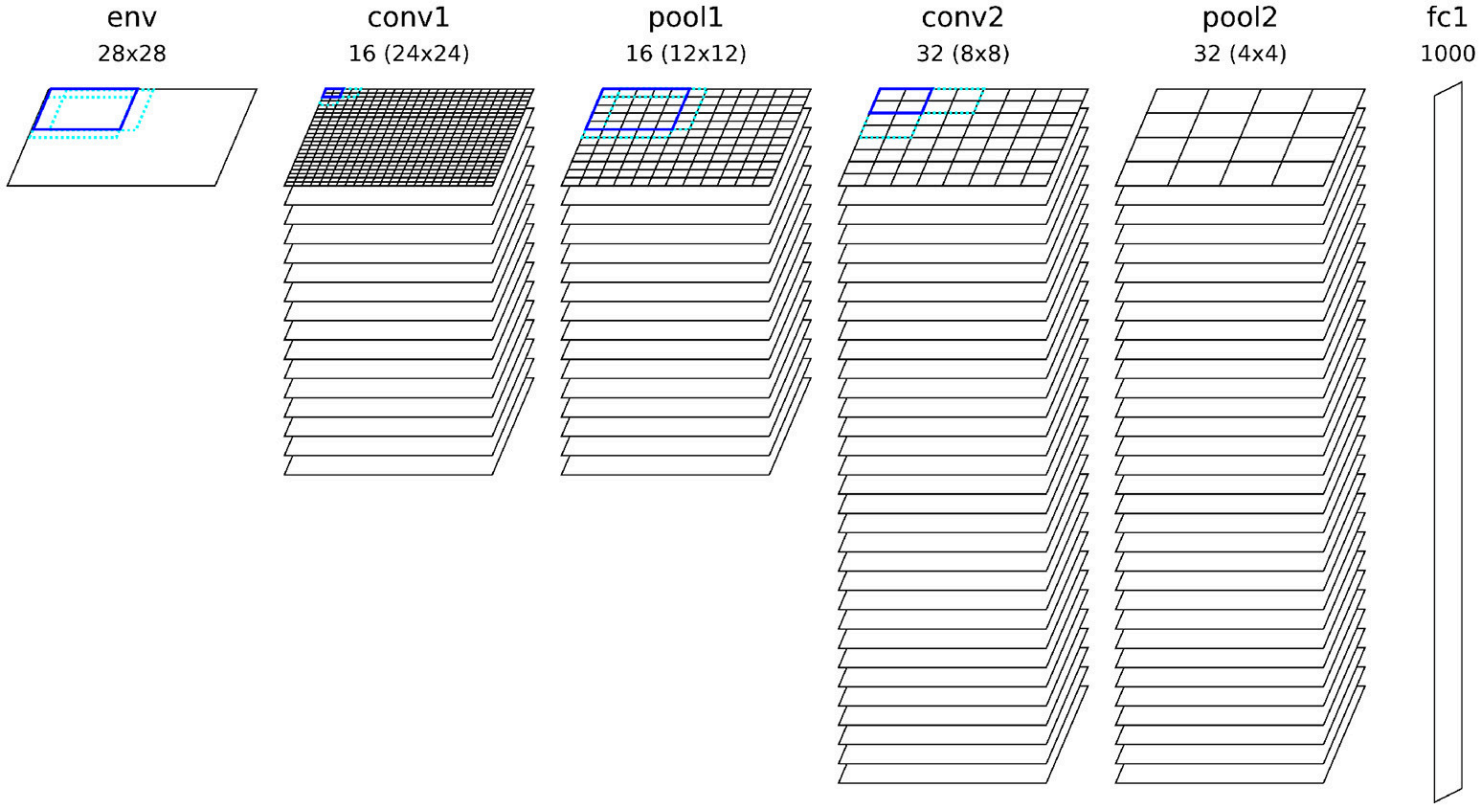
Configuration of the convolutional network architecture used for the experiment, showing all kernel sizes, strides and number of neural maps for each layer.

**Figure 4:**
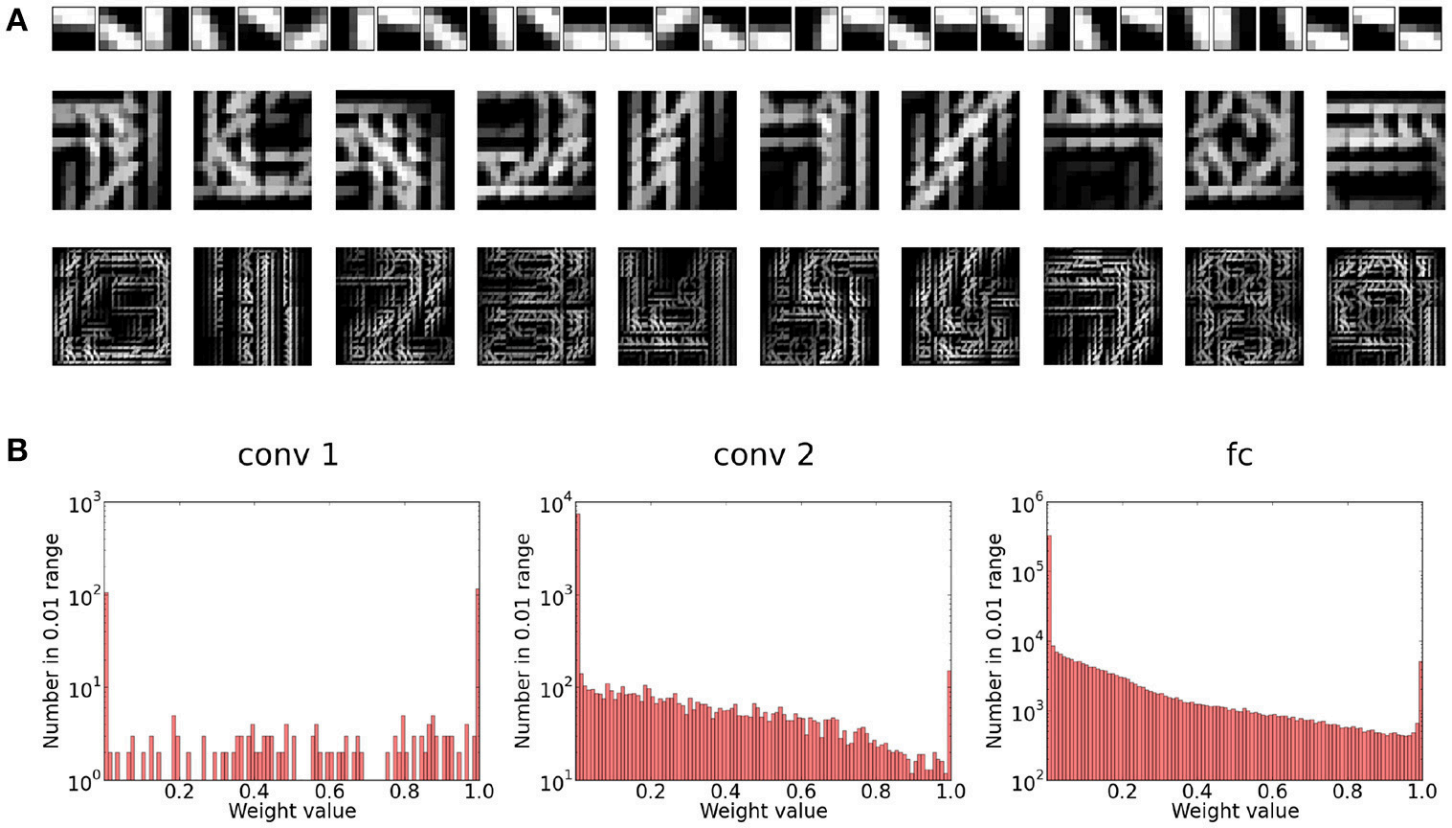
**(A)** Visualization of the preferred features learned in the different layers of the network. For the first layer, the preferred feature corresponds simply to the weight kernel. We can see that this layer learns filter patches which detect local contrast differences. The higher layer features are constructed by choosing for each neuron in the feature map the feature in the lower layer to which it has the maximum average connection strength. Note that due to the overlapping nature of the weight kernel of each position, the features have a somehow translational invariant appearance. As we can see for the second convolutional layer, the neurons become sensitive to parts of digits. Finally, in the fully connected layer, each neuron has learned a highly class specific version of a particular digit (digits 0–9 from left to right). **(B)** Weight distribution for the different layers of the network. Most weights converge to 1 or 0. In the higher layers, the weights become increasingly sparse.

**Figure 5:**
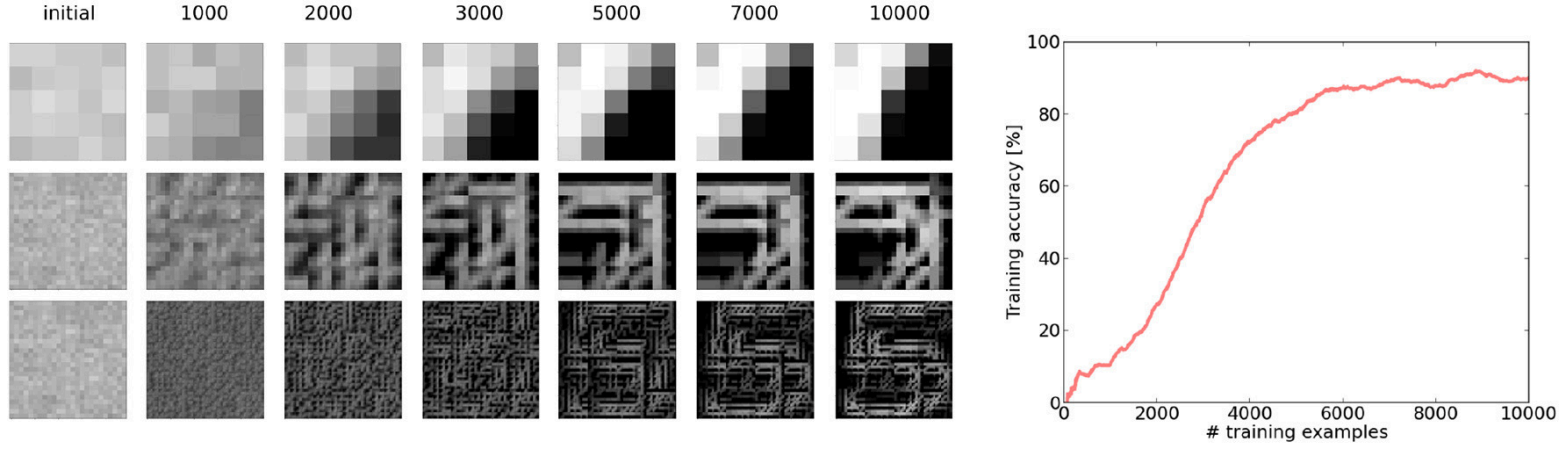
Demonstration of the simultaneous emergence of features in the different layers of the network. We can see that even if the features in the lower layers are not fully converged yet, the higher layer is able to assemble them to a more complex feature. The error plot shows the development of the running average error on the training set (averaged over the last 1,000 examples). We can see that even with the fuzzy features the top layer is able to perform an approximate inference, which continuously improves with the quality of the features.

Our first experiment tried to optimize the network for maximum performance on a single run of the full MNIST data set. The network configuration can be seen in Figure 3 and a visualization of the final features can be seen in Figure 4. As can be seen in Figure 5, all layers of the network do indeed converge simultaneously and classification performance increases continuously.

An interesting property of the final weight matrices is their sparsity and basically binary weight configuration. The sparsity of the weight matrices is caused by the sparse input and sparse responses of each layer due to the WTA mechanism. The binarization is caused by the STDP learning mechanism which enforces correlations. Synapses which receive consistently input that causes postsynaptic spikes will quickly saturate to the maximal weight value. During learning, this effect is attenuated by the exponential weight damping, which prevents a overly fast convergence of the weights to their maximal value. The fact that the final weights are almost binary leaves the possibility to binarize the weights fully after the learning phase without changing the representations significantly. Depending on the concrete hardware implementation, this would fully binarize the networks computations and may yield additional processing efficiency.

Even without this homeostatic mechanism and despite the similar learning rule, the maximal performance of our network (Figure **7**) is higher than for the network of Querlioz et al. (2015) for all neuron numbers in the fully connected layer. By increasing the number of features in the convolutional layers, the network is able to yield (96.58±0.16%) accuracy on the test set in the configuration with 16 and 256 maps in the convolutional layers. It is also better than the architecture of Diehl and Cook (2015), which uses besides a homeostatic mechanism several other mechanisms to stabilize learning and improve performance (such as divisive weight normalization, tuning of the input firing rates, and reset of neuron values for each example) as well as more synapses and iterations over the training set (4.2 Mio. adjustable synapses vs. 5 Mio. and 1 presentation vs. 15 presentations of the full MNIST data set). One main advantage of a convolutional architecture, as we have chosen it for this work, is that in contrast to shallow networks we can exploit translational invariance. Networks consisting only of a fully connected layer will fail to recognize a class if it is presented slightly shifted compared to the training set.

We also tested how strongly the online learning constraints affected the result. In the standard configuration, shown in Figure 3, the performance of the network is (95.2±0.2%). If we reset the neurons after each example presentation, the performance increases slightly but insignificantly to (95.24±0.26%). The same is true if we use a layer-wise training approach, where every layer is only trained on the full training set after the lower layers have been trained, which yields (95.27±0.23%) test set accuracy. We can thus conclude that our network does not seem to be significantly affected by these constraints relating to a database framework.

As other approaches which extract features with STDP and a winner-takes-all mechanism, the representations which are learned have some similarity with a k-means clustering algorithm. As it has been demonstrated in Coates et al. (2011), extracting features unsupervised with k-means and training a classifier on these features can yield good inference performance. However, modern deep learning architectures rarely make use of this unsupervised pretraining. One of the main reasons is that the type of features learned by such algorithms are so-called one-hot representations. This means that for a given input, only a few of the features will be activated, yielding a sparse code. On the other hand, representations learned by the backpropagation algorithm are typically distributed, which means that for a given input a lot of neurons will typically be activated and represent the input though their combined activity. In the parallel framework in which ANNs are typically used and with the ability to use high precision floating point numbers, distributed representations are usually superior since they are much more efficient from a coding perspective. In particular, GPUs can process very well the dense matrix multiplications which arise in this type of feature encoding. In a spiking network however, a high precision output of a neuron can only be obtained if we use a large number of spikes (at least as long as we use a rate code). In this case, the highly representative and sparse features of a one-hot representation enable us to encode complex information with much fewer spikes. Also, due to the event-based coding paradigm, a sparse code means that most inputs can be represented with only a few active neurons, while the other neurons remain completely silent (Kheradpisheh et al., 2017 showed that at most one spike per neuron per image can be sufficient). The price we have to pay for this sparsity is a kind of inefficient representation, which requires a large number of neurons (i.e., neural maps). We still think that it is a more suitable way to encode sparse representations in event-based systems. In such a system, neurons are only activated by external input and a large number of neurons does not necessarily produce a higher total activity, since most of the neurons will remain inactive. We can thus profit from sparse activity and its computational benefits even if the number of features (and therefore neurons) becomes very large.

### 3.2. Robustness to input variation and sparsity

In a follow-up experiment, we tested the robustness of learning to input presentation time variations. This feature is important for a real-world application, where we cannot be sure that all classes and objects will be presented to the network for the same fixed time. We therefore varied the presentation time of each digit randomly by a factor between 0.1 and 1.9, such that the total presentation time is on average equivalent to our other simulations. We observe that the final classification performance of the network seems to be insignificantly affected by these variations, yielding an accuracy of 95.13% compared to 95.33% with the same parameters and constant stimulus duration.

**Figure 6:**
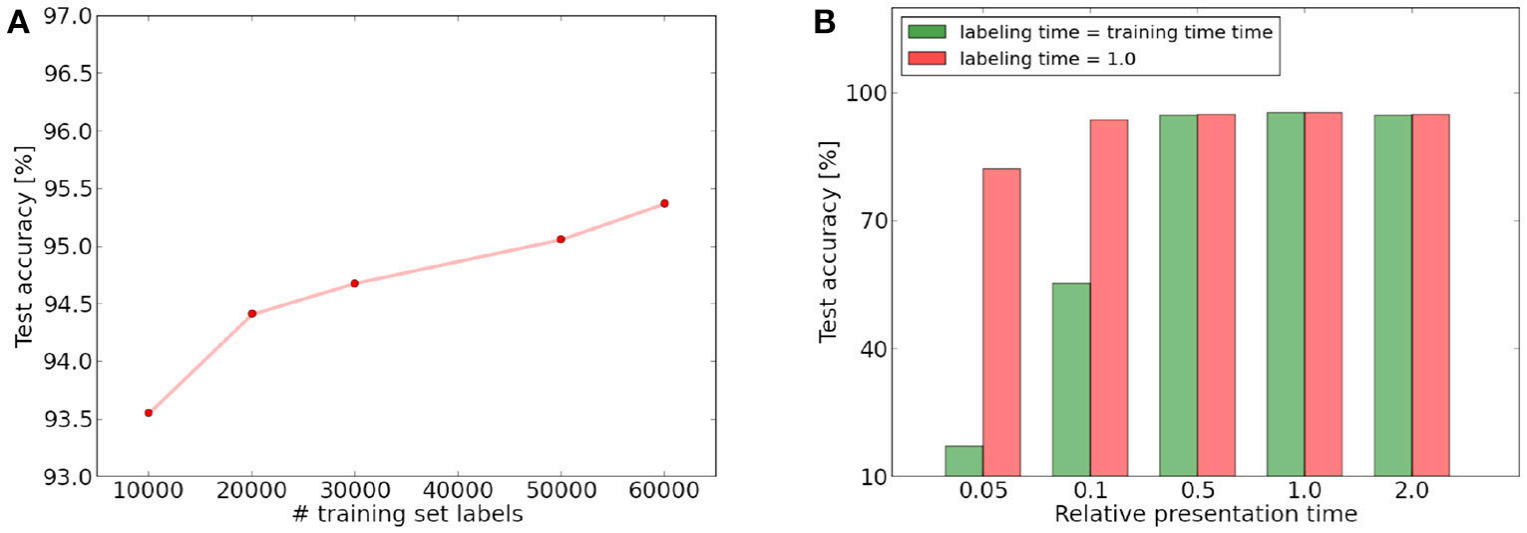
**(A)** Increase of test set performance as a function of the training set labels used to label the top layer neurons. **(B)** Influence of presentation time on training and labeling phase. Times are given relative to the presentation and labeling time we used for the other simulations. Green: test performance if presentation time during labeling and testing phase is same as during training. Red: performance if labeling and testing time is independent of the training phase presentation time and equal to the time represented by 1.0.

Additionally, we learned the whole training set with a constantly different presentation time and evaluated the performance (while adjusting the learning rates to account for the smaller number of spike events). We can see that as long as the presentation time stays within a certain range, the result is largely unaffected by the presentation time. This is in particular true for the feature learning, which seems to be mostly unaffected by the presentation time of a single image. We noted however that the presentation time is important for the inference phase. The performance of our spike count classifier drops significantly if the number of spikes triggered in the top layer becomes only a tenth of the original time (see Figure 6). If we use however the standard presentation time for the labeling and testing phase, the classification accuracy is still around 93.72%. It therefore seems that the drop in inference performance is mainly due to a failure of the classifier, which requires a certain number of spikes to label the neurons and classify correctly. This indicates that the network is able to learn features even with a presentation time per image which is one order of magnitude lower than the presentation time we chose for the other simulations. For this presentation time, there will be only approximately 100–500 input spikes for a single training example and the spikes triggered in the top layer are only in the order of 10. The total number of spikes triggered in the full network will be in the order of 1,000–2,000 depending on the image (if pooling neurons, input spikes and “pseudo-spikes” by the learning accumulators are discounted). For an image size of 28 × 28 = 784, this means that most pixels with a value significantly higher than 0 will spike only once or twice per examples and potentially very asynchronously. If we half the presentation time again to 5% of the original time, inference performance finally begins to drop strongly, although the feature learning mechanism still extracts meaningful features. For this presentation time, every images will be represented by only 40–100 spike events, which seems barely enough to give a meaningful representation of the 784 pixel digit. The drop in performance is therefore probably also caused by this discretization limit of the digit. Our results demonstrate that even with this asynchronous and sparse input, our architecture is able to extract useful features.

### 3.3. Scaling

**Figure 7:**
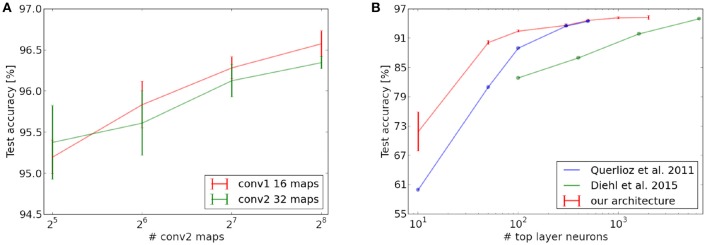
Scaling behavior of different layers of the network. **(A)** Scaling of the network performance with fixed number of top layer neurons and variable number of feature maps in the convolutional layers. **(B)** Increase in test set performance as a function of the number of neurons in the fully connected top layer (with number of feature maps fixed to 16 and 32 in the convolutional layers). The last point of Querlioz et al. (2015) is not exactly reported but likely lies between 94 and 95%.

We also analyzed the scaling behavior of our network, which is an important property for the potential of the architecture to be extended to more complex data. In a first experiment, we investigated how the size of the fully connected top layer affects the classification performance. Similar as Querlioz et al. (2011) and Diehl and Cook (2015), we could observe that an increase in the number of neurons in this layer leads to a higher classification accuracy. However, for our network, the performance increases substantially faster than for their architectures and our network yields higher or equal scores for all layer sizes (see Figure 7). This indicates that the pre-processing done by the convolutional layers indeed helps the fully connected layer to extract more general and useful digit prototypes, as compared to the case where the layer is directly connected to the input layer. Furthermore we trained on far less iterations over the MNIST set and did not use any homeostatic mechanisms in our fully connected layer. Querlioz et al. (2011) have observed in their work that such a mechanism substantially improves the performance of their network by balancing the competition between neurons. The fact that our architecture performs better even without this mechanism could indicate that the pre-processing of the convolutional layers produces a spiking output which is easier to process for the fully connected layer and increases the stability of the learning process.

In a next step, we also investigated the scaling properties of the convolutional layers by increasing the number of neural maps (see Figure 7), while leaving all other neuron variables untouched (in particular also the threshold of higher layers). Our results show that an increase in the number of maps in the second layer consistently leads to an increase in classification performance. This is not the case for the first convolutional layer. We suspect this could be caused by the relatively high redundancy between the maps of the first convolutional layer (see Figure 4). Since the competitive mechanism for the inference accumulators is rather weak, many similar maps can release a spike for the same input position and trigger a spike in the layer above (whose thresholds were not changed in the scaling process). This reduces the complexity of the features which can be learned in the higher layers and could therefore be responsible for the slight decrease in classification performance. Since the maps in the second convolutional layer are more complex, redundancy is lower and can be counterbalanced by the inter-map competition of the inference accumulators, which is why scaling seems to be more beneficial here.

It seems that our architecture can consistently profit from scaling, in particular in higher layers, where feature complexity is high. The competitive mechanism for learning will try to achieve maximal diversity for any number of maps while the inter-map competition of the inference accumulators can counterbalance potential redundancies in the propagation. Note that for both the fully connected and the convolutional layers we had to increase learning rates since the training time scales approximately linearly with the number of inhibited entities (i.e., maps for the convolutional layers and neurons for the fully connected one), which is a consequence of the winner-takes-all dynamics. This is only necessary to achieve convergence on a single presentation of the MNIST dataset and would not be a problem in an online learning setting, where unlabeled training images are abundant.

### 3.4. Robustness to learning rate variation

**Figure 8:**
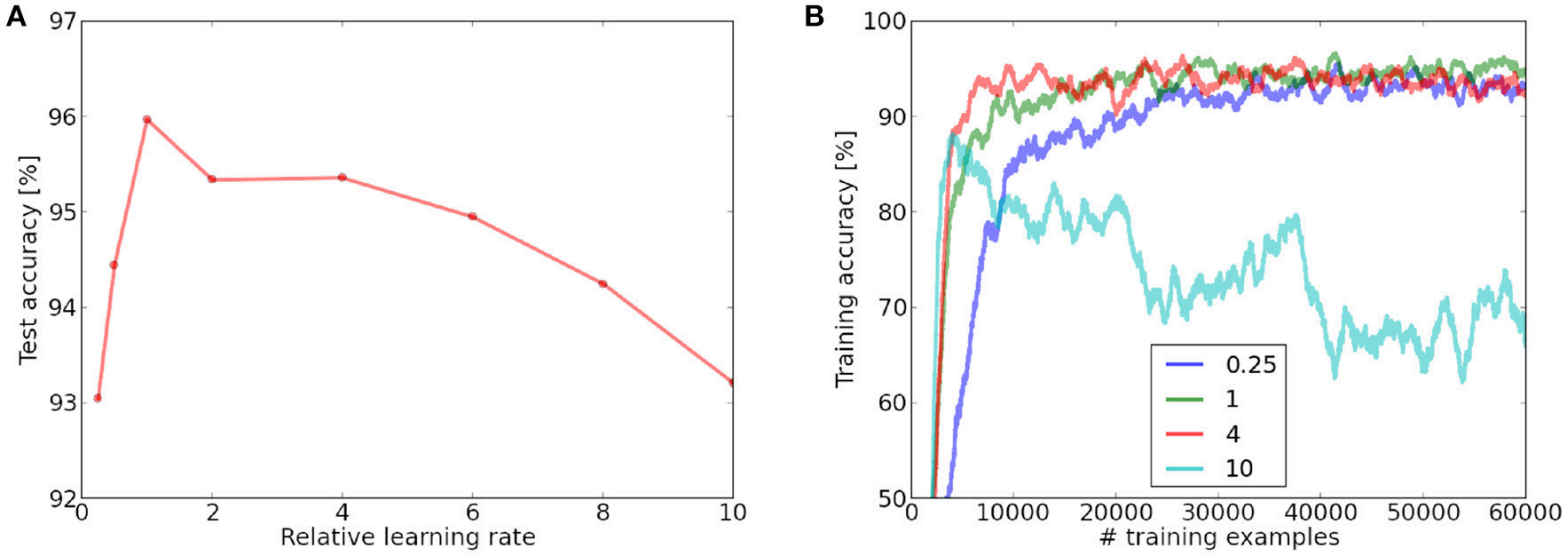
**(A)** Influence of the variation of the learning rate in the fully connected layer on the test performance, measured relative to the learning rate which enables convergence over a single dataset iteration. A learning rate which is too low will not converge over a single presentation of the MNIST dataset, while a very high learning rate leads to instabilities. **(B)** Online training performance for different learning rates (averaged over 1,000 last examples). Neurons are labeled online during the learning process.

While adjusting the learning rate for the scaling experiments, we observed that our architecture seems to be very robust to a change in the absolute values of the learning rates (while leaving the ratio between LTP and LTD constant). Figure 8 shows the inference performance on the last 1,000 examples of the training set and the test set as a function of the learning rate variation. Our experiments show that the network performance is remarkably stable with regard to the absolute value of the learning rates. In a value range for the learning rate spanning an order of magnitude, we can observe stable online training error convergence. A learning rate which is too low will not converge on a single presentation of the MNIST dataset (however if it is presented multiple times or if there would be more images). If the learning rate is very high, the online classification performance becomes unstable after an initial steep increase. This is probably mainly due to the online labeling mechanism which is used for classification. A high learning rate will alter the learned features of the neuron continuously and therefore the labeling mechanism fails. This could explain why the test performance is only affected to a comparably small extend, since in the test phase, neurons are labeled while learning is disabled.

In contrast to many ANN implementations, which are optimized for classification performance, we did not implement a learning rate variation policy, since this would violate the online learning paradigm. Our results show that even a constantly high learning can lead to robust convergence.

Depending on the specific application, the learning rate could be very high, allowing a fast adaption to changing input stimuli, or very low, which allows to include more information from training examples into the weights and therefore might lead to better generalization. We could also imagine a mechanism which changes the learning rate depending on the online classification performance. If the performance drops suddenly, the learning rate can be set to a higher value to let the network adapt to possibly unseen inputs.

Both the stability of the architecture for high learning rates and the promising scaling behavior have beneficial consequences for the parameter tuning process in a practical application. This is true in particular if the optimal architecture can not be found easily by an optimization process over a fixed training and test set. Initially, we could set the learning rates very high and use only a small number of neural maps to check if the neural maps quickly converge to a meaningful solution, which requires only a small unlabeled dataset with approximately the same properties as the online learning data. Such a solution could be easily identified given the intuitive local and hierarchical representations of the network and the ability to assess classification performance with only a few labels necessary for the spike-count classifier. If this is the case, the learning rates can be set to a low value and the network scaled up for the high precision online learning task.

## 4. Discussion and outlook

As other recent work in the field of spike-based learning rules, our work can be seen as a proof of concept, which demonstrates that multi-layer learning of hierarchical features with STDP is possible. Our work extends current approaches by its ability to train all layers simultaneously. This makes our architecture suitable for online learning in systems which receive a continuous stream of data from their environment and which have to perform learning and inference at the same time. Since the basic features of our architecture are not limited to any specific form of input, the network should be easily extendable to more complex natural images. As it has been shown in Kheradpisheh et al. (2017), the mechanisms of the architecture can be used to detect natural objects, such as faces or motorbikes.

Note that for the learning mechanisms used in this work, inference performance could possibly be optimized by a layer-wise training scheme as it was used in previous work. We refrained from using such a mechanism to demonstrate how a dual accumulator neuron can be used to provide sufficient input to higher layers even with an active inhibitory mechanism. The multi-layer nature of our architecture makes it accessible to learning mechanisms which involve multi-layer top-down feedback. In particular, the online predictions of our network could be used for a reinforcement learning scheme, which could modulate STDP learning with a reward signal which is propagated through the network. Additionally, multi-layer training is more compatible with an online learning paradigm, where it is not possible to receive a stimulus a second time. This could be problematic for a layer-wise training mechanism since higher layers would be trained on different inputs than the layers below. Finally, in contrast to other deep architectures which perform the feature extraction in the final layer with a more complex classifier to improve performance, the output of our network is highly specific and could be used directly for a higher level spiking processing stage (see for instance Eliasmith et al., 2012; Diehl and Cook, 2016 for functional spiking models of higher cortical processing).

As other systems trained purely with STDP, our network underperforms compared to current state-of-the-art CNNs, which easily yield classification accuracies beyond 99% (LeCun et al., 1998) on the MNIST benchmark. We want to emphasize here however that this difference in performance is to be expected since these networks are trained in a supervised fashion and with high precision activations (represented by floating point numbers). Their weights are therefore directly optimized to yield a high classification accuracy, which is not the case for an unsupervised architecture as the one we presented in this work, whose objective is merely to extract the statistically most relevent features of the training set given the constraints imposed by the architecture and the learning rules. As it has been shown recently by Neftci et al. (2017), an event-based version of backpropagation is able to yield performances much closer to frame based ANNs. The large differences in classification performances between networks trained with STDP and their frame based counterparts may therefore be caused to a large extent by differences in the training objective, and less by a general weakness of the event-based learning mechanism.

In contrast to the precise but resource hungry frame-based ANN approaches, our network is based on a spike code and therefore potentially very energy efficient if implemented on a neuromorphic hardware platform. Differences in timescale between inputs and hardware can be a problem for neuromorphic systems if they shall operate on natural stimuli in real time and the timescale of a neuromorphic system is often a design choice depending on the potential application (see for instance Qiao et al., 2015; Petrovici et al., 2017 for a real time and faster than real time analog neuromorphic hardware framework). Due to the fully event-based nature of our architecture, our network circumvents this problem and is able to operate on any time scales with computations being only driven by external input. Together with the simplicity of the neuron and synapse model, our architecture could be easily scaled up and implemented on a wide range of energy efficient neuromorphic hardware architectures for robotics and Internet-of-Things (IoT) applications. Its ability to extract features even from a strongly varying potentially sparse spiking input could make it suitable for feature extraction from a dynamic vision sensor (see for instance Lichtsteiner et al., 2008; Posch and Wohlgenannt, 2008).

## Author contributions

JT designed the architecture, performed the simulations, and wrote the manuscript. OB made conceptual contributions to the architecture and the simulation and gave feedback on the manuscript. AD provided feedback on architecture and manuscript.

### Conflict of interest statement

The dual accumulator neuron is protected under EU patent EP17203159 “A STDP-Based Learning Method for a network having dual accumulator neurons. The authors declare that the research was conducted in the absence of any commercial or financial relationships that could be construed as a potential conflict of interest.
